# Transcriptomic and Lipidomic Analysis Reveals Complex Regulation Mechanisms Underlying Rice Roots’ Response to Salt Stress

**DOI:** 10.3390/metabo14040244

**Published:** 2024-04-21

**Authors:** Yingbin Xue, Chenyu Zhou, Naijie Feng, Dianfeng Zheng, Xuefeng Shen, Gangshun Rao, Yongxiang Huang, Wangxiao Cai, Ying Liu, Rui Zhang

**Affiliations:** 1 College of Coastal Agricultural Science, Guangdong Ocean University, Zhanjiang 524088, China; ybx@gdou.edu.cn (Y.X.); zhou_chenyu444@163.com (C.Z.); fengnj@gdou.edu.cn (N.F.); zhengdf@gdou.edu.cn (D.Z.); shenxuefeng@gdou.edu.cn (X.S.); raogs518@126.com (G.R.); gdouhrb@126.com (Y.H.); 2 South China Branch of National Saline-Alkali Tolerant Rice Technology Innovation Center, Zhanjiang 524088, China; 3 College of Chemistry and Environment, Guangdong Ocean University, Zhanjiang 524088, China; m13715805122@163.com

**Keywords:** transcriptome, lipidomics, rice, salt stress

## Abstract

Rice (*Oryza sativa* L.), a crucial food crop that sustains over half the world’s population, is often hindered by salt stress during various growth stages, ultimately causing a decrease in yield. However, the specific mechanism of rice roots’ response to salt stress remains largely unknown. In this study, transcriptomics and lipidomics were used to analyze the changes in the lipid metabolism and gene expression profiles of rice roots in response to salt stress. The results showed that salt stress significantly inhibited rice roots’ growth and increased the roots’ MDA content. Furthermore, 1286 differentially expressed genes including 526 upregulated and 760 downregulated, were identified as responding to salt stress in rice roots. The lipidomic analysis revealed that the composition and unsaturation of membrane lipids were significantly altered. In total, 249 lipid molecules were differentially accumulated in rice roots as a response to salt stress. And most of the major phospholipids, such as phosphatidic acid (PA), phosphatidylcholine (PC), and phosphatidylserine (PS), as well as major sphingolipids including ceramide (Cer), phytoceramide (CerP), monohexose ceramide (Hex1Cer), and sphingosine (SPH), were significantly increased, while the triglyceride (TG) molecules decreased. These results suggested that rice roots mitigate salt stress by altering the fluidity and integrity of cell membranes. This study enhances our comprehension of salt stress, offering valuable insights into changes in the lipids and adaptive lipid remodeling in rice’s response to salt stress.

## 1. Introduction

Rice (*Oryza sativa* L.) is one of the most important crops on earth and is the main diet of 50% of the world’s population [1]. The area of rice is more than 150 million hectares; however, most agricultural areas are partly salinized or at risk of salinization [2]. Rice is a salt-sensitive crop, as salt stress reduces the grain yield of rice by more than 50% [3,4]. At the seedling stage, rice is vulnerable to salt stress [5] and it becomes highly susceptible during the reproductive stage [6]. However, rice demonstrates relative tolerance during seed germination [7] and the early vegetative stage, which includes shoot and root development until the emergence of the first tiller [8]. Breeding salt-tolerant rice is crucial for food security, as it can help us cope with current and potential food crises. To achieve this, it is essential to explore the mechanism of salt stress in rice to develop salt-tolerant genotypes.

The response mechanisms of salt tolerance in plants involve osmotic adjustment, reactive oxygen species (ROS) scavenging, maintenance of ionic homeostasis, and nutritional balance [9]. While ROS serves as a signal in response to salt stress, excess ROS, such as the superoxide anion (O_2_^•−^), singlet oxygen (^1^O_2_), the hydroxyl radical (·OH), and hydrogen peroxide (H_2_O_2_), can induce oxidative stress [10]. This oxidative stress can react with vital biomolecules, causing a range of damage, such as lipid peroxidation, protein denaturation, and DNA mutation. To prevent the buildup of ROS, plants have evolved defense mechanisms. These include promoting the biosynthesis and accumulation of compatible osmolytes, increasing the activity of antioxidant enzymes such as catalase (CAT), peroxidase (POD), and superoxide dismutase (SOD), as well as non-enzymatic antioxidants such as ascorbic acid (ASA), alkaloids, carotenoids, flavonoids [11], and glutathione (GSH) [9]. Many antioxidant enzyme genes regulate salt tolerance in plants. Accumulation of ROS stimulates a mitogen-activated protein kinase (MAPK) cascade, which activates enzymatic antioxidants [12,13]. MAPK regulates specific cellular responses by modifying the expression of the target proteins or transcription factors. However, as the salt concentration increases, the antioxidant system can become overwhelmed, unable to remove excess ROS on its own, resulting in the rapid accumulation of O_2_^•−^ and H_2_O_2_. The increases in the malondialdehyde (MDA) content and membrane lipid peroxidation cause rice cells to suffer membrane damage, ultimately affecting normal growth and development [14].

Maintenance of ionic homeostasis is another strategy for salt tolerance. Salt stress impacts numerous genes. The SOS pathway, which is a fundamental and evolutionarily conserved signal transduction route, plays a pivotal role in removing excessive Na^+^ out of the cells. SOS1 is a plasma membrane-localized Na^+^/H^+^ antiporter, which is regulated by the SOS2–SOS3 complex (SOS2, a serine/threonine protein kinase; SOS3, a calcium-binding protein) to expel Na^+^ out of the cell. HIS1-3, a histone linker protein, negatively regulates the SOS pathway through the transcriptional regulation of the SOS genes, while the TF WRKY1 oppositely regulates them [15]. PLATZ2 inhibits the transcription of SOS3 and SCaBP8, both of which are crucial for SOS2’s kinase activity [16]. Using transcriptome high-throughput sequencing technology, it has been found that the HKT and NHX family genes differ in salt tolerance when comparing barley and rice transcriptomes [17]. The vacuolar Na^+^/H^+^ antiporters OsNHX1, OsNHX2, OsNHX3, OsNHX4, and OsNHX5 were reported to maintain the compartmentalization of Na^+^ and K^+^ [18,19]. The high-affinity K^+^ transporter (HKT) family mainly functions to maintain Na^+^/K^+^ homeostasis in the cytoplasm. In addition to the HKT and NHX family genes, the genes regulating HKT, NHX, and CLC also play important roles in salt tolerance, such as OsMYBc, OsbZIP71, and OsNF-YC13 [9].

Maintaining the membrane’s stability and integrity under salt conditions is a crucial adaptation to salt stress, as it safeguards the cell’s homeostatic balance [20,21]. The plant cell membrane, serving as the primary semi-permeable barrier, plays a pivotal role in protecting the cell from the disruptive effects of salt stress [22,23]. The main structural components of the plasma and endomembrane are lipids, including phospholipids, glycolipids, glycerides, fatty acids, and sphingolipids [24]. Under salt stress, plants demonstrate adaptations in the accumulation, composition, and saturation of lipids to cope with the stressful environment [25]. The accumulation of phospholipids in response to salt stress has been well documented through numerous studies. PAleon, a recently developed PA-specific biosensor, has been used to observe the accumulation of PA during salt stress. It was observed that PA accumulates rapidly within 10 min of salt treatment, primarily in the roots [26]. PA activates the Na^+^/H^+^ antiporter SOS1, promoting Na^+^ efflux by binding to MPK6 and stimulating its kinase activity, which phosphorylates SOS1 [27], or binding to the residue Lys57 in SOS2, enhancing the activity and plasma membrane localization of SOS2, leading to the activation of SOS1 [28]. Additionally, PA promotes the phosphorylation of the SOS3-like calcium-binding protein 8 (SCaBP8) by SOS2 under salt stress, diminishing SCaBP8-mediated inhibition of *Arabidopsis* K^+^ transporter 1 (AKT1) [28].

The addition of exogenous PC significantly improves the tolerance of annual peach tree (*Prunus persica* (L.) Batsch.) to salt stress and mitigates its damage [29]. PC can interact with ACBP, enhancing plants’ salt tolerance by increasing PLDδ’s activity and further converting PC to PE, PS, and PG to stabilize the cell membrane [30]. Furthermore, salinity induces the accumulation of PS [20,31]. PS regulates salt stress tolerance by regulating the activity of PM H^+^-ATPas, the PM Na^+^/H^+^ antiporter, and maintaining ion homeostasis. Additionally, it may even regulate the electrostatic field of the plasma membrane [32]. Overexpression of the sphingosine-1-phosphate (S1P) lyase gene has been shown to decrease salt tolerance in tobacco [33]. Conversely, the overexpression of the ceramide (Cer) catalase gene increases the salt tolerance of *Arabidopsis thaliana* [34]. Interestingly, the ectopic expression of *GhIPCS1* (inositol phosphatidyl ceramide (IPC) synthase) in cotton leads to increased IPC content and heightened sensitivity to salt stress [35]. These lipid-mediated adaptations allow plants to maintain membrane stability and integrity under salt conditions, thus enhancing their tolerance to environmental stress.

The salt tolerance mechanism of rice has been extensively examined, yet the impact of lipids on this mechanism remains under-explored. For this study, a conventional rice variety, Huanghuazhan, was selected. The primary aim of this investigation was to conduct a combined lipidomic and transcriptomic analysis to pinpoint the changes in the membrane’s lipid metabolism and delve into the regulation of lipid remodeling in rice roots under salt stress. Unraveling the regulatory patterns of lipid metabolism in rice during salt stress sets the stage for a deeper understanding of the regulation mechanism of lipid metabolism, ultimately aiding in safeguarding crop productivity and optimizing the efficient utilization of saline land.

## 2. Materials and Methods

### 2.1. Plant Materials and Growth Conditions

The rice cultivar, Huanghuazhan, was used in this study. Seeds were surface-sterilized with a 5% sodium hypochlorite solution for 15 min and rinsed several times with distilled water. Then the rice seeds were germinated in a dish covered with filter paper for 4 days. Seedlings with consistent growth were moved to a 96-well hydroponic box filled with Yoshida hydroponic nutrient solution with (salt treatment) or without (CK) 50 mM NaCl for 14 days. Three groups of replicates were set up. The nutrient solution was refreshed every 3 days. At 14 days, the shoots and roots were harvested for measurement.

### 2.2. Phenotypic Measurement

For the measurement of plant height, root length, and dry weight, CK and NaCl treatment samples were obtained from three biological replicates, and each biological replicate contained 20 plants. Root length was scanned with a WinRHIZO LA6400XL root scanner and the data were analyzed with WinRHIZO Pro 2005b software (Régent Instruments Inc., Quebec, QC, Canada).

### 2.3. Analysis of Physiological and Biochemical Indices 

Measurement of superoxide dismutase (SOD), peroxidase (POD), and catalase (CAT) activity and proline (Pro) content was made, following the methods of Gu et al. [36]. The malondialdehyde (MDA) content was determined by the thiobarbituric acid method according to Yan et al. [37]. For leaf chlorophyll (Chl) content, the relative values of the third leaves were measured using a chlorophyll meter (SPAD-502Plus, Konica-Minolta Company, Osaka, Japan).

### 2.4. RNA Sequencing and Analysis

To study the changes in whole transcripts of rice roots in response to salt stress, RNA-Seq analysis was conducted as described previously [38]. The total RNA of roots from the control and NaCl treatments was extracted. Each treatment had three biological replicates. After treatment with DNase I, the complementary DNA was synthesized to construct the cDNA library using the AMPure XP system. Sequencing was performed on an MGISEQ-T7 platform. After rigorous filtering to eliminate low-quality sequences, the clean reads were then mapped to the reference genome. The gene expression levels were estimated as the fragments per kilobase per million (FPKM) mapped. The gene expression levels with |log2foldchange| > 1 and *p* < 0.05 were identified as the differentially expressed genes (DEGs) between the CK and NaCl treatments. Functional enrichment was comprehensively evaluated utilizing both the Gene Ontology (GO) framework and the Kyoto Encyclopedia of Genes and Genomes (KEGG) database.

### 2.5. RNA Extraction, Reverse Transcription, and Real-Time Quantitative PCR

Total RNA extraction was performed using the TaKaRa MiniBEST Plant RNA Extraction Kit (Takara, Bio, Tokyo, Japan). The total RNA was stored at −80 °C. About 1 µg of RNA was treated with DNase I, then the reverse transcription kit was used to synthesize the first-strand complementary DNA. The primers used for qPCR are listed in Table S1. The OsActin gene was used as a reference. The real-time quantitative polymerase chain reaction (RT-qPCR) was conducted using a CFX Connect™ Real-Time PCR System (Bio-Rad, Hercules, CA, USA), and the relative expression levels were calculated using the 2^−∆∆CT^ method [39]. Each gene had three biological replicates.

### 2.6. Lipid Extraction and UHPLC-MS/MS Analysis

The same materials were used for both the lipidomic and the transcriptomic analysis. For the lipidomic analysis, six replicates were processed. Lipid extraction and mass spectrometry analysis were performed by Shanghai Applied Protein Technology. The specific methods were described in detail by Zhu et al. [38]. Quantitative results were based on the ion signal’s intensity. LipidSearch v4.0 software (Thermo Scientific, San Jose, CA, USA) was used for lipid analysis, in which lipids with VIP > 1 and *p*-value < 0.05 were considered to be differentially accumulated metabolites.

### 2.7. Data Analysis

The data were expressed as mean ± standard error (SEM). All data were processed and underwent statistical analysis using Excel 2019 and IBM SPSS Statistics V25.0 for one-way analysis of variance (ANOVA), followed by Duncan’s multiple range tests; *p* < 0.05 was considered significant. The histograms were generated with Origin 2022.

## 3. Results

### 3.1. Response of Growth and Photosynthetic Properties of Rice under Salt Stress

**Figure 1:**
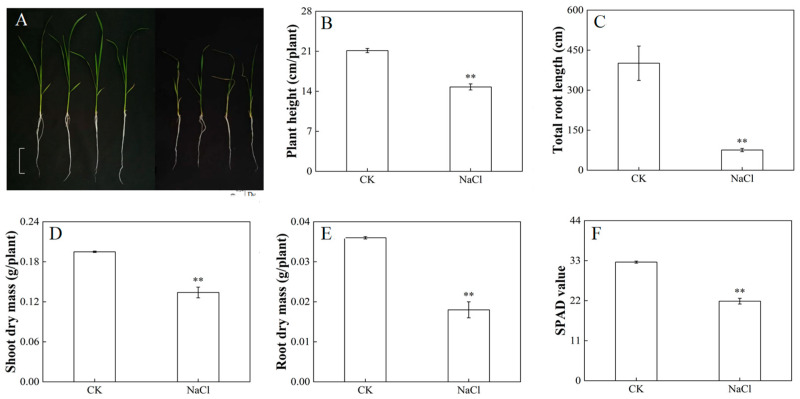
Effect of salt stress on the phenotype and photosynthetic properties of rice. (**A**) Phenotype of rice, bar = 5 cm. (**B**) Plant height. (**C**) Total root length. (**D**) Shoot dry mass. (**E**) Root dry mass. (**F**) SPAD value. Experimental data are expressed as the mean and standard deviation (SD) of three biological replicates. Significance analysis was performed using the Waller–Duncan model. “**” indicates a highly significant difference at the *p* < 0.01 level.

After 14 days of NaCl treatment, the effect of salt stress on the growth of rice was explored (Figure 1). The salt-treated rice showed significant yellowing and wilting of the leaves, and shortened roots (Figure 1A). Rice under salt stress had a significant decrease in plant height (Figure 1B) and total root length (Figure 1C), which decreased by 30.13% and 81.1%, respectively, compared with rice cultured under normal conditions. Under salt stress, the biomass of the shoot (Figure 1D) and the root (Figure 1E) was significantly lower than that of rice under normal conditions. Accordingly, the chlorophyll content (Figure 1F) of rice under salt stress significantly decreased compared with CK. As the main organ of plants that absorbs nutrients from the outside, the root system plays a key role in plants’ growth and development. Roots are the first organs to be exposed to the soil, so they are also the first ones to perceive salt stress and are the most vulnerable to salt stress. In the morphology of the root system, the overall length of the roots was significantly reduced, and the number of lateral roots and the root absorption area were significantly reduced (Figure 1A,C).

### 3.2. Response of the Physiological Properties of Rice under Salt Stress

**Figure 2:**
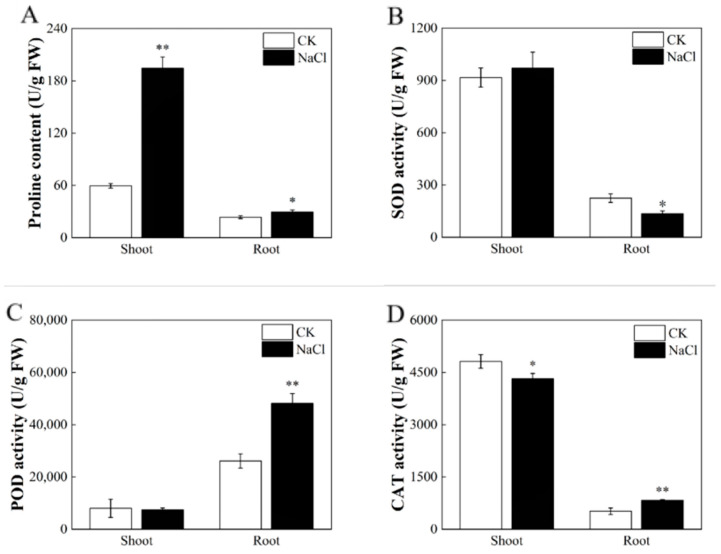
Effect of salt stress on the physiological characteristics of rice plants. (**A**) Proline content; (**B**) SOD activity; (**C**) POD activity; (**D**) CAT activity. Experimental data are expressed as the mean and standard deviation (SD) of three biological replicates. Significance analysis was performed using the Waller–Duncan model. “*” indicates a significant difference at the *p* < 0.05 level and “**” indicates a highly significant difference at the *p* < 0.01 level.

Rice suffers osmotic stress caused by salt stress. In order to reduce this damage, plants accumulate organic solutes and inorganic solutes to protect against osmotic damage, among which, the most sensitive osmotic regulatory material is proline. In this study, the proline content was significantly affected in different parts of rice seedlings under salt stress. In the shoots, the proline content exhibited a significant increase of 218.51% compared with the control group (CK), as depicted in Figure 2A. Similarly, the proline levels in the roots were notably distinct from the control, with an increase of 23.91% (Figure 2A).

Rice produces excessive ROS under salt stress and causes membrane oxidative damage. The antioxidant enzymes, such as catalase (CAT), peroxidase (POD), and superoxide dismutase (SOD), reduce the content of reactive oxygen species in plants. The activities of SOD, POD, and CAT in the roots were detected. The results revealed that the activities of SOD decreased by 39.71% in salt-treated seedlings in contrast to the control (Figure 2B). The activity of POD increased by 84.44% (Figure 2C) and activity of the CAT increased by 61.29% (Figure 2D).

### 3.3. Changes in the Transcriptomes in Rice Roots Resulting from NaCl Stress

**Figure 3:**
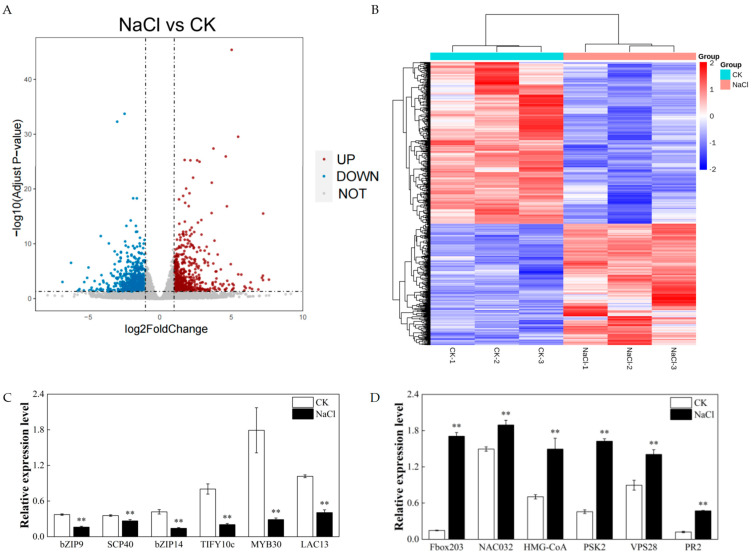
Transcriptomic analysis of NaCl and CK samples based on RNA-seq data. (**A**) Volcano plot of the differential expression analysis. (**B**) DEGs of all samples obtained by applying k–means clustering. (**C**,**D**) Expression levels of target gene obtained by RT-qPCR: bZIP9 (LOC_Os05g39540), SCP40 (LOC_Os07g46350), bZIP14 (LOC_Os02g03960), TIFY10c (LOC_Os09g26780), MYB30 (LOC_Os02g41510), LAC13 (LOC_Os05g38390), Fbox203 (LOC_Os04g31120), NAC032 (LOC_Os02g56600), HMG-CoA (LOC_Os01g16350), PSK2 (LOC_Os11g05190), VPS28 (LOC_Os01g57260), and PR2 (LOC_Os05g31140). Significance analysis was performed using the Waller–Duncan model. “**” indicates a highly significant difference at the *p* < 0.01 level.

To analyze the responsive genes in rice roots when subjected to salt stress, RNA-seq transcriptome analysis was conducted on rice roots grown under the CK and NaCl treatments. The results showed that about 57,512,451 and 54,030,857 raw reads were obtained in CK and NaCl roots, respectively (Table S2). Among the raw reads, about 57,501,566 and 54,017,240 were clean reads from the CK and NaCl treatments, respectively (Table S2). More than 70% of the clean reads were mapped to the rice reference genome (Table S2). Furthermore, 29,127 genes were identified expressed in roots under the CK or NaCl treatment (Table S3). Compared with the CK, 1286 differentially expressed genes (DEGs), including 526 upregulated genes (log2foldchange > 1 and Padj < 0.05) and 760 downregulated genes (log2foldchange < −1 and Padj < 0.05), were found in roots from the NaCl treatment (Figure 3A,B and Table S4).

RT-qPCR was used to detect the expression levels of 12 identified transcriptome (RNA-seq) DEGs to validate the RNA-seq data (Figure 3C,D). Twelve DEGs were selected from the previous results, which were associated with response of rice to salt stress. The results of RT-qPCR were positively correlated with those of RNA-seq. Six of the twelve genes selected after salt stress were significantly higher than those of the control treatment (Figure 3C), and the other six genes were significantly lower than those from the transcriptome sequencing analysis (Figure 3D).

**Figure 4:**
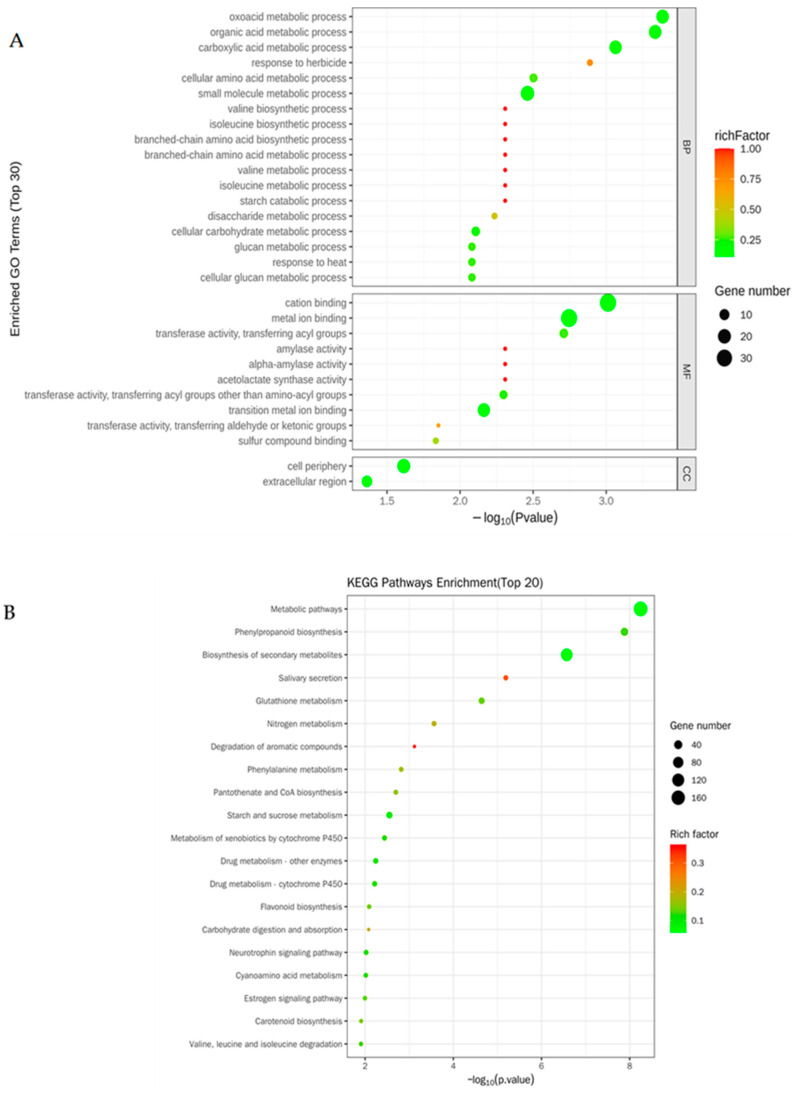
GO and KEGG analyses of the DEGs. (**A**) GO enrichment analysis of the DEGs. (**B**) KEGG enrichment analysis of the DEGs.

Gene Ontology (GO) enrichment analysis of these DEGs was also conducted, and the top 30 enriched terms were divided into three parts, including biological processes (BP), molecular functions (MF), and cellular components (CC), as shown in Figure 4A. For the biological processes, there were 21 DEGs involved in small-molecule metabolic processes, 18 DEGs involved in oxoacid metabolic processes, 18 DEGs involved in organic acid metabolic processes, 17 DEGs involved in carboxylic acid metabolic processes, 7 DEGs involved in cellular carbohydrate metabolic processes, and 6 DEGs involved in cellular amino acid metabolic processes (Figure 4A). For the molecular functions, there were 39 DEGs involved in cation binding; 7 DEGs involved in transferase activity, transferring acyl groups; 3 DEGs involved in sulfur compound binding; 2 DEGs involved in amylase activity; and 2 DEGs involved in acetolactate synthase activity (Figure 4A). For the cellular components, there were 19 DEGs involved in the cell periphery and 11 DEGs involved in the extracellular region (Figure 4A).

Kyoto Encyclopedia of Genes and Genomes (KEGG) analysis of these DEGs was further conducted, and the top 20 enriched pathways are shown in Figure 4B. The results showed that the most enriched pathway was the metabolic pathway, including 162 DEGs, followed by the biosynthesis of secondary metabolites, including 104 DEGs (Figure 4B). There were 32 DEGs enriched for phenylpropanoid biosynthesis (Figure 4B). The number of DEGs that were enriched for glutathione metabolism, and starch and sucrose metabolism were the same, which was 16 (Figure 4B). There were nine DEGs enriched for the metabolism of xenobiotics by cytochrome P450; eight DEGs enriched for nitrogen metabolism, seven for phenylalanine metabolism; six for valine, leucine, and isoleucine degradation; six for flavonoid biosynthesis; five for carotenoid biosynthesis; and four for carbohydrate digestion and absorption (Figure 4B).

### 3.4. Expression of Key Genes Associated with Lipids under Salt Stress

**Figure 5:**
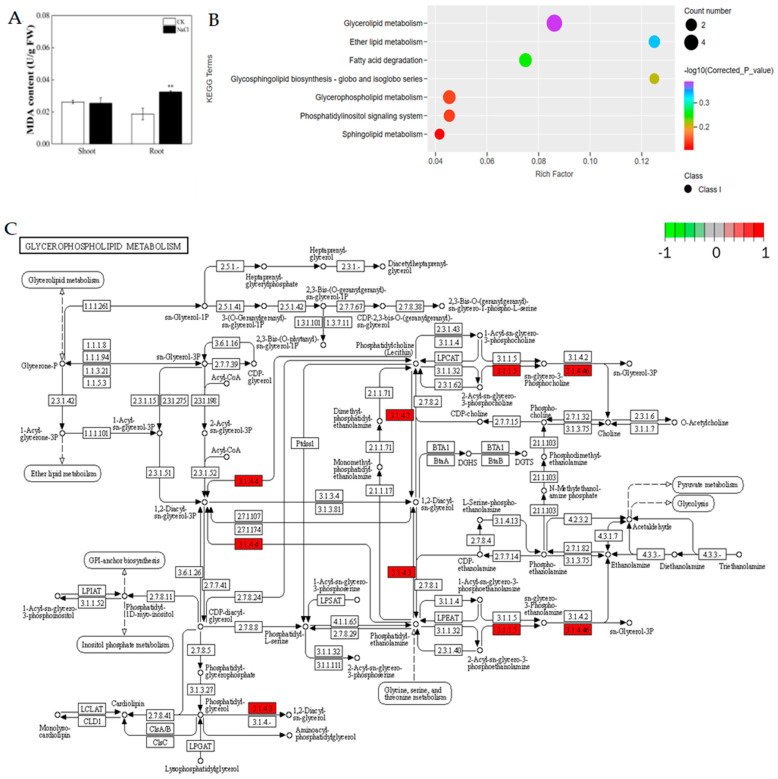
Effects of lipid peroxidation in rice without or with 50 mM NaCl. (**A**) The content of MDA in rice roots and leaves. Significance analysis was performed using the Waller–Duncan model. “**” indicates a significant difference at the *p* < 0.01 level. (**B**) KEGG enrichment analysis of the DEGs related to lipids. (**C**) DEGs involved in glycerophospholipid metabolism. Circles represent metabolites. The boxes between metabolites indicate the enzymes that catalyze the reaction between the two metabolites. Red indicates that the DEGs encoding the enzyme were upregulated, and green indicates that the DEGs encoding the enzyme were downregulated. 3.1.4.4, phospholipase D (LOC_Os01g07760); 3.1.4.3, non-specific phospholipase C (LOC_Os03g63580); 3.1.1.5, lysophospholipase (LOC_Os04g57380); 3.1.4.46, glycerophosphodiester phosphodiesterase (LOC_Os07g41150).

The membrane is a lipid bilayer structure and is the primary target for the damage induced by salt stress [32]. To resist salt stress, several alterations in the structure and functionality of the cell membrane occurred. The MDA content was detected. The results showed that the MDA content of rice roots treated with 50 mM NaCl was significantly higher than that in the control plants (Figure 5A). As the main structural components of the plasma and endomembrane are lipids, to gain an insight into the gene expression levels of lipid metabolism genes and decode the transcriptional regulatory network under salt stress, a KEGG analysis of DEGs related to lipids was performed (Figure 5B). The results indicated that the transcriptome levels of glycerophospholipid metabolism, the phosphatidylinositol signaling system, and sphingolipid metabolism were involved in salt stress (Figure 5B). Among them, there were four DEGs enriched in glycerophospholipid metabolism including a *phospholipase D* (encoding EC3.1.4.4), a non-specific phospholipase C (encoding EC3.1.4.3), a lysophospholipase (encoding 3.1.1.5), and a glycerophosphodiester phosphodiesterase (encoding EC3.1.4.46), and all of them were upregulated by the NaCl treatment (Figure 5C). The phospholipase D participated in the hydrolyzation of phosphatidylcholine and phosphatidylethanolamine to generate 1,2-diacyl-sn-glycerol-3P (Figure 5C). The non-specific phospholipase C participated in the hydrolyzation of phosphatidylcholine, phosphatidylethanolamine, and phosphatidylglycerol to generate 1,2-diacyl-sn-glycerol (Figure 5C). The lysophospholipase participated in the hydrolyzation of 2-acyl-sn-glycero-3-phosphocholine and 2-acyl-sn-glycero-3-phosphoethanolamine to generate sn-glycero-3-phosphocholine and sn-glycero-3-phosphoethanolamine, respectively (Figure 5C). The glycerophosphodiester phosphodiesterase participated in the hydrolyzation of sn-glycero-3-phosphocholine and sn-glycero-3-phosphoethanolamine to generate sn-glycero-3P (Figure 5C).

### 3.5. Response of the Lipid Composition of Rice under Salt Stress

**Figure 6:**
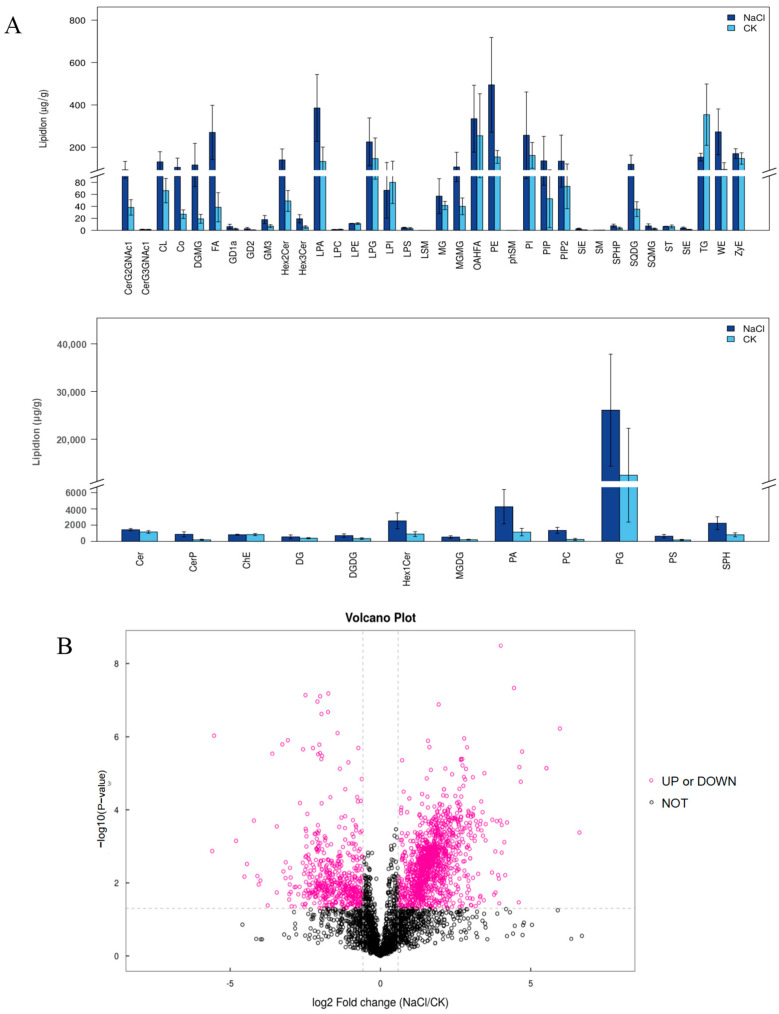
Lipidomic analysis in the roots of rice. (**A**) The changes in 48 categories of lipids between the NaCl and CK groups. (**B**) Volcano plot of different molecules between NaCl and CK. Pink dots indicate significant changes, while gray dots indicate no significant changes.

To study the effects of salinity on the changes in the intracellular lipid content, a lipidomic analysis of rice roots was performed. In total, 3542 identified lipid molecules were detected in rice roots with or without salt stress, including 48 lipid subclasses (Figure 6A,B and Table S5). The lipid subclass composition of the samples from each group is presented in ring plots, as shown in Figure S1. The top lipid subclasses in CK were PG, Cer, PA, Hex1Cer, and ChE (Figure S1A), while PG, PA, Hex1Cer, SPH, and Cer were the main lipid components of the samples under the NaCl treatment (Figure S1B). Among them, the most significant changes in the lipid profile in response to salt stress were observed in phospholipids, as illustrated in Figure 6A. The total lipid content of PA, PC, PS, Cer, CerP, Hex1Cer, and SPH showed a significant increase under salt stress, while the triglyceride (TG) content showed significant decreases following salt treatment (Figure 6A). In total, 249 lipid molecules with VIP > 1 and *p*-value < 0.05 were identified as differentially accumulated lipid metabolites (DAMs) (Table S6). Heat maps were constructed, and hierarchical clustering analysis of the DAMs was conducted, and most of the differentially expressed lipid metabolites were found to increase in response to salt stress. DAMs clustered in the same cluster had similar expression patterns (Figure S2).

### 3.6. Response of the Lipid Chain Length and Chain Saturation under Salt Stress

**Figure 7:**
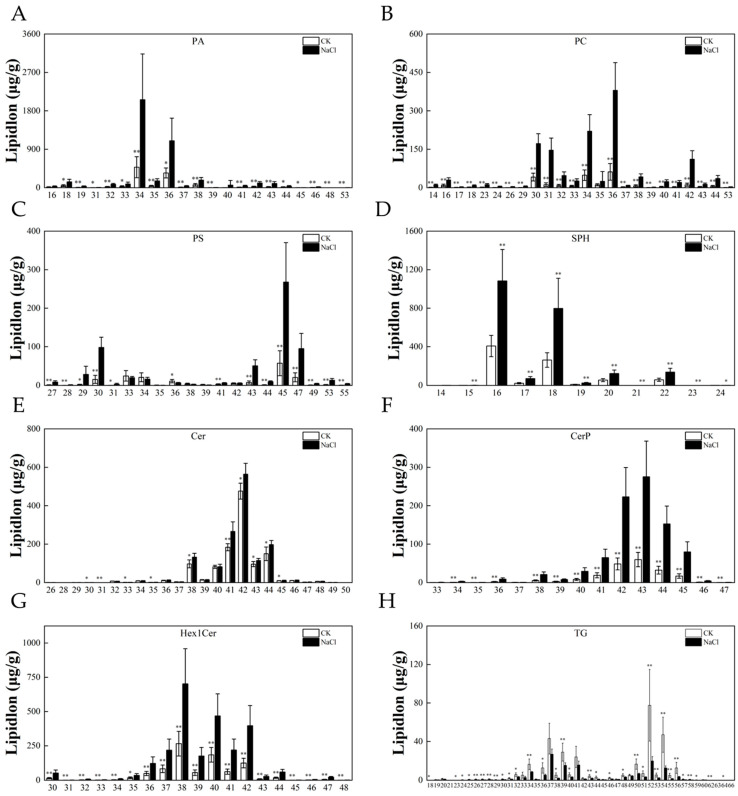
Analysis of lipid chains’ length. Differences in the content of lipid molecules with different carbon chain lengths: (**A**) PA, (**B**) PC, (**C**) PS, (**D**) SPH, (**E**) Cer, (**F**) Cerp, (**G**) Hex1Cer, and (**H**) TG. Experimental data are expressed as the mean and standard deviation (SD) of three biological replicates. Significance analysis was performed using the Waller–Duncan model. “*” indicates a significant difference at the *p* < 0.05 level and “**” indicates a highly significant difference at the *p* < 0.01 level.

**Figure 8:**
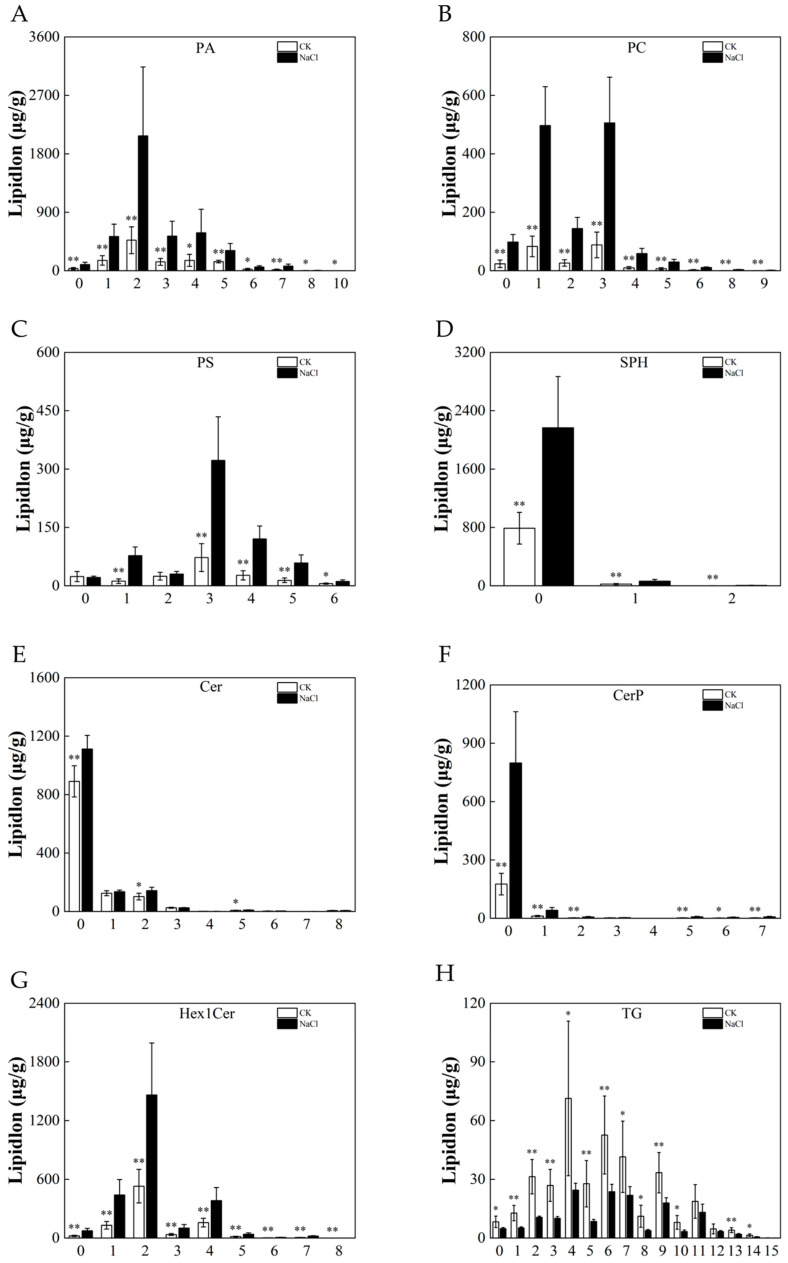
Analysis of lipid chains’ saturation. Differences in the content of lipid molecules with different numbers of unsaturated bonds: (**A**) PA, (**B**) PC, (**C**) PS, (**D**) SPH, (**E**) Cer, (**F**) Cerp, (**G**) Hex1Cer, and (**H**) TG. Experimental data are expressed as the mean and standard deviation (SD) of three biological replicates. Significance analysis was performed using the Waller–Duncan model. “*” indicates a significant difference at the *p* < 0.05 level and “**” indicates a highly significant difference at the *p* < 0.01 level.

The chain length and saturation of lipids are also important factors affecting lipids’ functions. In addition to the content of lipids and the function of lipids, chain length affects the thickness of the cell membrane, the membrane’s mobility, and the activity and function of target proteins. The double bonds introduce kinks, reduce the packing density of acyl chains, and inhibit the change in the cell membrane from a fluid to a solid phase gel state. To further analyze the effects of lipid chains’ length and the degree of unsaturation on salt stress, we analyzed the differences in eight subclasses. This showed that salt stress led to the elongation of chain length in the roots and an increase in the chains’ saturation in rice (Figure 7 and Figure 8).

### 3.7. Multi-Omics Combined Analysis of Rice Seedlings’ Roots in Response to Salt Stress

**Figure 9:**
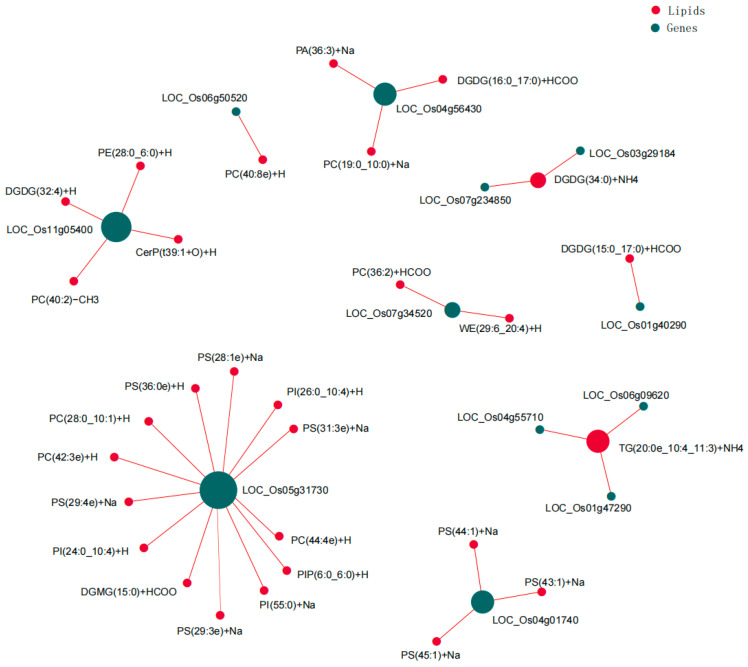
Network plot of the correlation analysis of significant differential genes with significant differential lipids. In the figure, the green circles represent the lipids with significant differences, the red circles represent the genes with significant differences, and the thickness of the lines is proportional to the absolute value of the correlation coefficient. The size of a node is positively correlated with its degree of connectivity (Degree), that is, the greater the connectivity, the larger the node’s size.

Correlation network analysis refers to the use of the correlation coefficient to establish a network’s interactions, providing a new perspective on the correlation between genes with significant differences and lipids with significant differences. The elements of the correlation need to have a certain connection or relationship before the correlation analysis. According to the Pearson correlation coefficient, we could measure the degree of association between the genes and lipids in the sample. To screen for lipids with significant differences at key nodes in the network and genes with significant differences, Pearson’s correlation network analysis of the top 50 genes and top 50 lipids with significant differences with a correlation coefficient value |r| of ≥0.5 and *p* < 0.01 was performed using Cytoscape (version 3.7.0) software. In total, 29 significantly correlated differential genes and 12 differential lipids, including 32 correlated pairs, identified 9 nodes (Figure 9).

## 4. Discussion

It is well known that rice is a salt-sensitive crop. Germination and the early seedling stages are considered to be sensitive stages in the plant’s life cycle [5,35]. Salt stress seriously affects the growth, development, yield, and quality of rice. Salt stress decreases relative growth by decreasing the efficiency of photosynthesis, reducing rice stands’ density, limiting seedlings’ production of biomass, and inhibiting the elongation and proliferation of cells [40]. In addition, we found that the accumulation of biomass decreased significantly (Figure 1), while the tolerance index decreased relative to the control, which was consistent with the results of previous relevant studies [41]. This study documented the inverse relationship between chlorophyll content and salt concentration levels (Figure 1F), indicating that exposure to salt stress slowed down photosynthesis and chlorophyll synthesis, thus affecting plants’ growth and performance [42].

Osmotic and ion stress induced by salt stress can damage plants’ cells and metabolic pathways, thus affecting their growth and development [43]. Various inherent mechanisms exist in plants to counter the breakdown of major metabolic pathways due to salt stress. Among these mechanisms, the antioxidant enzyme system plays the most critical role in clearing overproduced ROS and maintaining plant cells’ homeostasis under stress conditions [44]. Major players in this antioxidant enzyme system include SOD, POD, and CAT. In this study, the activities of POD (Figure 2C) and CAT (Figure 2D) in rice roots increased with the salt stress, showing cellular protection and antioxidant behavior. When plants are exposed to salt stress, plant cells may lack water due to internal and external ion imbalance, leading to water shortages, which is known as osmotic stress. Under osmotic stress, plant cells regulate osmosis by increasing solutes, which usually have osmotic activity and can increase the concentration of cells and thus reduce the cells’ osmotic potential. These solutes are called osmoregulators, and their presence ensures normal water supply to the plant under osmotic stress and maintains normal plant growth and development [45]. In our experiment, higher proline activity and antioxidant activities (Figure 2) were indicators of salt stress resistance in rice.

In this study, we analyzed DEGs (Figure 3 and Figure 4) and the differential metabolites of lipid metabolic pathways (Figure 6) in rice roots under long-term salt stress, indicating that most of the reactions in the endoplasmic reticulum synthesis pathway and sphingolipid synthesis pathway were activated, and most of the genes that catalyze these reaction steps were upregulated under salt stress. A recent report showed that the level of PC in corn roots decreased under salt stress, which was opposite to the results of this study on the level of PC in rice roots (Figure 6A), indicating that salt stress activated the renewal of PC and lipid reprogramming [46]. PA can be produced by the PLD and PLC diacylglycerol kinase (DGK) pathways [47]. In the PLD pathway, PA can be produced by the hydrolysis of membrane phospholipids such as PC and PE [47,48]. In the PLC-DGK pathway, the phospholipid hydrolyzed membrane in PLC produces diacylglycerol (DAG), which is then converted to PA through a DGK-catalyzed phosphorylation reaction [49]. It can be seen from the results of this study that PA decreased with the accumulation of salt stress (Figure 6A and Table S6), indicating that PA played a regulatory role in the related signal transduction. There are two common methods of PS biosynthesis in plants: one is to use cytidine diphosphate (CDP-DAG) and serine as substrates to catalyze the synthesis of PS by CDP-DAG-dependent PS synthase (CD-PSS). Another uses serine and phospholipids (e.g., PC and PE) through an exchange reaction between the phospholipid’s head and serine, with Ca^2+^ being catalytically dependent on the base exchange PSS (BE-PSS) [50]. PS is involved in salt stress tolerance by regulating the activity of PM H^+^-ATPase, the activity of the PM-Na^+^/H^+^ antiporter, ion homeostasis, and even the electrostatic field of the plasma membrane. In rice, researchers found that the PS produced by PS synthase is a lipid molecule necessary for internode cell elongation and the exocytosis pathway in rice [51]. Therefore, an increase in PS content can effectively promote exocytosis and plasma membrane repair. In this study, the PS content of the root system of Huanghuazhan rice was significantly increased (Figure 6A and Table S6). We hypothesized that from the germination stage to the trifoliate stage, salt stress caused cell damage, and in order to prevent further damage, the cells began to initiate repair mechanisms.

So far, there have been few reports on the changes in and effects of sphingolipids under salt stress. *Arabidopsis* neuraminidase, AtACER, hydrolyzes ceramides to SPH and FA. At the same time, atacer mutants downregulated the expression of AtACER while transgenic *Arabidopsis thaliana* showed increased salt sensitivity during seed germination and root growth. However, overexpression of the AtACER gene significantly improved the salt tolerance of transgenic *Arabidopsis* plants [34]. Therefore, it was speculated that the increase in the sphingolipid content (Figure 6A) may positively regulate the salt tolerance of rice. In light of this, it is reasonable to guess that the sphingolipid content will increase significantly after long-term salt stress, which is consistent with the results obtained in this study using Huanghuazhan rice.

The main lipids in plants are glycerides, in which the carboxyl group of fatty acids is linked to the hydroxyl ester of glycerol. Lipid synthesis involves multiple organelles in the cell. Fatty acids are synthesized by the chloroplasts and combine directly with glycerol to form galactolipids, which are the main component of the chloroplast membrane. The fatty acids are transferred to the cytoplasm and bind to the glycerol in the endoplasmic reticulum (ER) to become the phospholipids of the cell membrane [52]. In the endoplasmic reticulum of the seed cells, triglycerides (TG) are synthesized and stored in the oil body [53]. Therefore, TG is a storage lipid in plants and plays an important role under various stress conditions [54,55]. A large amount of TG is also accumulated in aging leaves [56]. In this study, it was found that the root system of Huanghuazhan after long-term salt stress contained a small amount of TG compared with the control system (Figure 6A). We speculate that salt stress can potentially disrupt cellular structures, including organelles such as the endoplasmic reticulum, which are responsible for lipid synthesis, thereby affecting the synthesis and storage of triglycerides (TG).

In this study, we found that after Huanghuazhan rice was exposed to salt stress from germination, signs of aging were observed in the three-leaf monophyllum stage; repair of the plasma membrane was slow; and PC, PA, PS, Cer, CerP, Hex1Cer, and TG enabled the membrane to play an important role in maintaining stability and fluidity under salt treatment conditions. Future studies will further explore the specific roles of individual lipid species and their interactions with other cellular components in rice’s response to salt stress. Additionally, it would be interesting to investigate whether genetic engineering or agronomic practices can be used to enhance rice’s tolerance to salt stress by manipulating lipid metabolism or other related pathways.

## 5. Conclusions

In this study, we aimed to investigate the effects of salt stress on the physiological quality, gene expression, and lipid diversity of rice seedlings. Through a comprehensive analysis combining transcriptomic and lipidomic approaches, we identified 1286 differentially expressed genes, indicating alterations in the gene expression patterns in response to salt stress. Moreover, our lipidomic analysis revealed significant changes in the composition and unsaturation of lipids, with significant increases in the major phospholipids and sphingolipids, and a sharp decrease in triglyceride levels. Collectively, our results suggest that rice roots mitigate salt stress by altering the fluidity and integrity of cell membranes, possibly through changes in the composition and metabolism of lipids. This study enhances our comprehension of salt stress responses in rice and offers valuable insights into changes in the lipid and adaptive lipid remodeling.

In conclusion, this study provides new insights into the physiological and molecular mechanisms underlying rice’s response to salt stress, highlighting the importance of lipid remodeling in salt tolerance. Our findings have implications for improving rice production in salt-affected areas and contribute to the ongoing efforts to ensure food security in the face of environmental challenges.

## Data Availability

The data presented in this study are available on request from the corresponding author due to legal reasons.

## Supplementary Materials

The following supporting information can be downloaded at https://www.mdpi.com/article/10.3390/metabo14040244/s1. Table S1. Primers used in this study. Table S2. Statistical summary of the reads from RNA-seq. Table S3. General information of all the identified genes. Table S4. General information of the DEGs. Table S5. Results of the qualitative and quantitative analyses of lipid molecules. Table S6. General information om the differentially accumulated metabolites. Figure S1. Composition of lipid subclasses. Figure S2. Heat map and hierarchical clustering analysis of the differentially accumulated lipid metabolites.
