# Supplementary material for: Transcriptomic and Lipidomic Analysis Reveals Complex Regulation Mechanisms Underlying Rice Roots’ Response to Salt Stress
Source: Metabolites. 2024 Apr 21;14(4):244. doi: 10.3390/metabo14040244 (PMC11052231; doi:10.3390/metabo14040244)
Supplement: Supplementary file 1 [file metabolites-14-00244-s001.zip › Supplementary Figures-4.15.pdf]

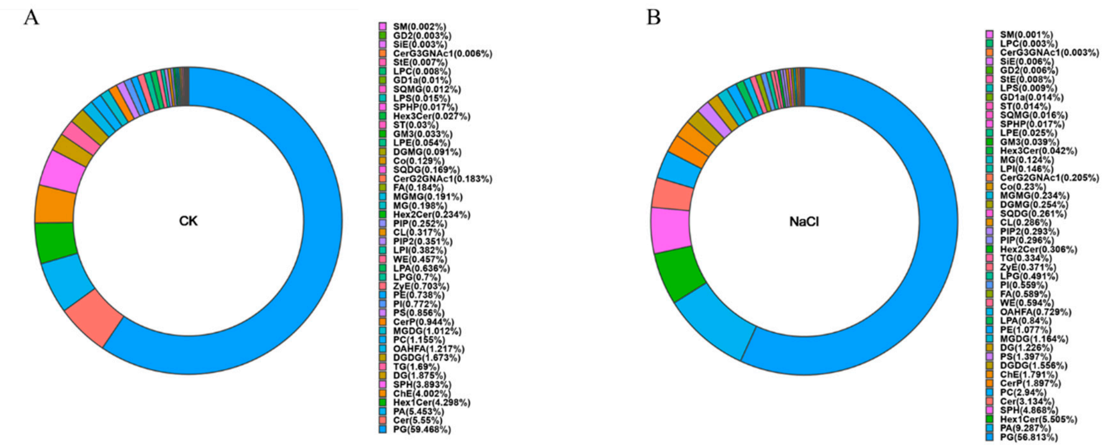

Figure S1. Lipid subclass composition analysis of rice root under CK (A) and NaCl (B) treatments . Different lipid subclasses are represented by different colors, and the proportion is represented by the size of the color block area

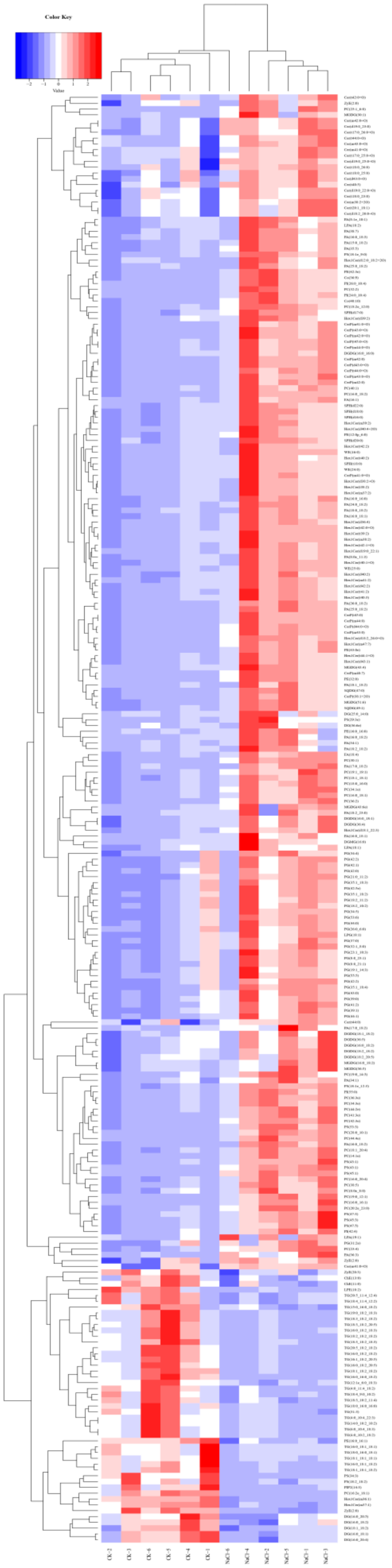

Figure S2. Heat map and hierarchical clustering analysis of the differentially accumulated lipid metabolites.
